# Influenza vaccination among healthcare workers in a multidisciplinary University hospital in Italy

**DOI:** 10.1186/1471-2458-8-422

**Published:** 2008-12-23

**Authors:** Susanna Esposito, Samantha Bosis, Claudio Pelucchi, Elena Tremolati, Caterina Sabatini, Margherita Semino, Paola Marchisio, Francesco della Croce, Nicola Principi

**Affiliations:** 1 Institute of Pediatrics, University of Milan, IRCCS Ospedale Maggiore Policlinico, Mangiagalli e Regina Elena, Milan, Italy; 2 Department of Epidemiology, Istituto di Ricerche Farmacologiche Mario Negri, Milan, Italy; 3 Health Management Unit, Fondazione IRCCS "Ospedale Maggiore Policlinico, Mangiagalli e Regina Elena", Milan, Italy

## Abstract

**Background:**

Annual influenza vaccination is recommended for healthcare workers (HCWs) in order to reduce the morbidity associated with influenza in healthcare settings. The aim of this study was to evaluate the current vaccination status of the HCWs in one of Italy's largest multidisciplinary University Hospitals.

**Methods:**

Between February 1 and March 31, 2006, we carried out a cross-sectional study of influenza vaccination coverage among HCWs at the University Hospital Fondazione IRCCS "Ospedale Maggiore Policlinico, Mangiagalli e Regina Elena", Milan, Italy. After receiving a brief description of the aim of the study, 2,143 (95%: 1,064 physicians; 855 nurses; 224 paramedics) of 2,240 HCWs self-completed an anonymous questionnaire.

**Results:**

Influenza vaccination coverage was very low in all specialties, varying from 17.6% in the Emergency Department to 24.3% in the Surgery Department, and knowledge of influenza epidemiology and prevention was poor. The factors positively associated with being vaccinated were an age of ≥ 45 years, considering influenza a potentially severe disease, and being aware of the high-risk categories for which influenza vaccination is strongly recommended; those that negatively associated with being vaccinated were being female, working in the Medicine Department, and being a nurse or paramedic.

**Conclusion:**

Despite strong recommendations, influenza vaccination coverage seemed to be very low among HCWs of all specialties, with differences between areas and types of employment. Specific continuous educational and vaccination programs for different targets should be urgently organized to reduce morbidity and mortality in high-risk patients, contain nosocomial outbreaks, and ensure an appropriate socioeconomic impact.

## Background

Influenza is a major health problem, and a significant clinical and socioeconomic burden in all age groups [1-3]. Serious illness and death rates are highest among the elderly (those aged ≥ 65 years), children aged <2 years, and people of any age with a medical condition that places them at increased risk for related complications [4-6]. Influenza vaccination is the most effective means of preventing influenza virus infection and its potentially severe complications [7], and efforts to promote immunization are mainly directed toward providing vaccinations for people at risk for influenza complications and their contacts.

The United States Advisory Committee on Immunization Practices (ACIP) and health authorities throughout the world recommend annual influenza vaccinations for healthcare workers (HCWs) in order to reduce the morbidity associated with influenza in healthcare settings [7-10]. This recommendation is based on evidence showing that the vaccination of HCWs can significantly reduce patient morbidity and mortality, and that outbreaks of hospital-based influenza occur where unvaccinated HCWs are employed [11-13]. The vaccination of HCWs has been associated with reduced absenteeism and financial savings for healthcare institutions [14,15], whereas some authors have found that HCWs tend to work during their illnesses, thus putting their patients at risk [16].

However, although the vaccination of HCWs is a high priority everywhere [8-10], data from various surveys show that fewer than 50% are actually vaccinated in different countries [17-24]. The reluctance of HCWs to accept influenza vaccination is associated with lack of knowledge of influenza and its complications, older age, having employer-provided healthcare insurance, or having visited a healthcare professional during the previous year [17-24].

There were no exhaustive and comprehensive data on compliance with influenza vaccine recommendations in Italy. We have recently published a paper on the attitudes towards influenza vaccination among the staff of Italy's largest Department for the Health of Women and Children, which were investigated in order to verify compliance with national guidelines and the possibility of adopting US recommendations for pregnant women and children [24]. We found that the respondents had little knowledge of influenza prevention, which was also demonstrated by the fact that very few HCWs vaccinated their patients [24]. However, this previous study was focused mainly on understanding whether obstetricians/gynecologists, neonatologists and pediatricians were convinced that influenza was a relevant problem to their patients and actively promoted influenza vaccination. No information on job categories as well as specialties different from HCWs caring for women and children were available. The aim of this study was to evaluate the current vaccination status of the HCWs in all of the Departments different from the Department for the Health of Women and Children of one of Italy's largest multidisciplinary University Hospitals, as well as their knowledge of and attitudes towards influenza vaccination.

## Methods

### Participants

Between February 1 and March 31, 2006, we carried out a cross-sectional study of influenza vaccination coverage among HCWs at the University Hospital Fondazione IRCCS "Ospedale Maggiore Policlinico, Mangiagalli e Regina Elena", Milan, Italy, as well as their knowledge of and attitudes towards influenza vaccination. The hospital has a Medicine Department with 13 wards (Allergy, Cardiology, Dermatology, Endocrinology, Immunology, Gastroenterology, Geriatrics, Hematology, Internal Medicine, Nephrology, Neurology, Pneumatology, Psychiatry), a Surgery Department with eight wards (Opthalmologic Surgery, General Surgery, Neurosurgery, Otorhinolaryngology, Thoracic Surgery, Transplant Surgery, Urology, Vascular Surgery), an Emergency Department with four wards (Intensive Care, Emergency Medicine, Emergency Surgery, Intensive Neurologic Care) and a Services Department with 11 Units (Anatomic Pathology, Biochemistry Laboratory, Microbiology, Transfusion Laboratory, Transplant Laboratory, Health Management, Neuroradiology, Nuclear Medicine, Radiology, Radiotherapy, Pharmacy).

During the study period, the involved Departments employed 2,240 HCWs (1,590 females, 70.9%) with a median age of 39 years (range 19–68), including 1,110 physicians (49.6%), 891 nurses (39.8%), and 239 (10.7%) paramedics (health aides/healthcare assistants). There were 910 employees (40.6%) in the Medicine Department, 440 (19.6%) in the Surgery Department, 346 (15.4%) in the Emergency Department, and 544 (24.3%) in the Services Department.

Over the last ten years, all of the HCWs have been offered influenza vaccinations free of charge albeit without any specific educational information campaign being organised, and they were also free to receive the influenza vaccine from other sources.

### Study questionnaire

After receiving a brief oral and written description of the aim of the study, all of the participants received a self administered standardized questionnaire to complete at pre-arranged times. All of the HCWs on the wards at the time of the morning or afternoon change of shift were invited to participate.

The anonymous questionnaire, which was different from the one used in our previous study [23], was developed iteratively by experts in general medicine, surgery, emergency and preventive medicine, and public health, addressed multiple domains, and was pilot-tested on a convenience sample of physicians, nurses and paramedics in order to ensure clarity and ease of administration. It consisted of five items and 18 questions including age and gender, their personal use of influenza vaccination, their perception of the seriousness of influenza, and their general knowledge of influenza recommendations and preventive measures. It has to be self-completed in 20 minutes in a separate room by all HCWs.

The study was approved by the Ethics Committee of the Institutional Review Board of the Fondazione IRCCS "Ospedale Maggiore Policlinico, Mangiagalli e Regina Elena", Milan, Italy. Written informed consent was obtained from all of the participants before study entry.

### Statistical analysis

Descriptive statistics of the responses were generated. Categorical data were analyzed by χ^2 ^test, whereas continuous data were analyzed by *t *tests for independent samples. All of the analyses were two tailed, and *p *values of 0.05 or less were considered significant.

Odds ratios (ORs) and their 95% confidence intervals (CIs) were calculated using unconditional multiple logistic regression, in order to measure the associations between HCWs' characteristics, knowledge and attitudes and vaccination status. Covariates were selected *a priori*, on the basis of previous knowledge on the issue [24-26] and personal experience. The multivariate model included terms for age, gender, department, type of employment, attitude towards seasonal influenza, influenza illness during previous year, number of offspring and awareness of all of the high-risk categories for which influenza prevention is strongly recommended. Those variables that were not associated (all p-values > 0.05) with vaccination status were not presented. All of the analyses were made using SAS software, version 8.2 (Cary, NC).

## Results

The questionnaires were fully completed by 2,143/2,240 (95.7%) HCWs, 1,500 (69.9%) of whom were female; their median age was 39 years (range 19–68): 895 (41.8%) worked in the Medicine Department (520 physicians, 285 nurses, 90 paramedics), 413 (19.3%) in the Surgery Department (177 physicians, 177 nurses, 59 paramedics), 337 (15.7%) in the Emergency Department (131 physicians, 158 nurses, 48 paramedics), and 498 (23.2%) in the Services Department (236 physicians, 235 nurses, 27 paramedics). Ninety-seven HCWs (15 from the Medicine Department, 25 from the Surgery Department, 11 from the Emergency Department, and 46 from the Services Department) refused to participate because they thought it would take too long to complete the questionnaire (n = 49) or they were not convinced of the real benefit of the project (n = 48).

Table 1 shows the influenza vaccination coverage of the HCWs, and their reason for getting vaccinated or not. There was a 100% cumulative response to each question. Coverage was very low in all specialties, ranging from 17.6% of the HCWs in the Emergency Department to 24.3% of those in the Surgery Department. Regardless of their specialties, the majority of the HCWs received their vaccination from the hospital prevention service. The main reason for vaccination was the fear of transmitting the disease to their patients, although a majority of the HCWs in the Emergency Department stated that the fear of transmitting influenza to their families was the most important. The main reason for not getting vaccinated was the absence of a fear of the disease in all groups of respondents. No differences in the reasons for getting vaccinated or not were observed according to job category. Moreover, the different wards in each Department showed similar reasons. Only a minority of the HCWs in each group had received at least one influenza vaccination.

**Table 1:** Influenza vaccination coverage among healthcare workers, and their reasons for undergoing influenza vaccination or not.

Questions and answers	Overall results (No. = 2,143)	Medicine (No.= 895)	Surgery (No.= 415)	Emergency (No.= 335)	Services (No.= 498)
Did you receive influenza vaccination this year?					

Yes	432 (20.2)	175 (19.6)	101 (24.3)*	59 (17.6)	97 (19.4)

Who gave you influenza vaccination this year?					

The hospital prevention service	354/432 (81.9)	141/175 (80.6)	82/101 (81.2)	49/59 (83.0)	82/97 (84.5)

A vaccination center near my Home	13/432 (3.0)	6/175 (3.4)	3/101 (3.0)	1/59 (1.7)	3/97 (3.1)

Medical friends	65/432 (15.1)	28/175 (16.0)	16/101 (15.8)	9/59 (15.3)	12/97 (12.4)

Why do you undergo influenza vaccination?					

Because I am a HCW	40/432 (9.3)	15/175 (8.5)	8/101 (7.9)	7/59 (11.9)	10/97 (10.3)

Because I am afraid of transmitting influenza to my patients	152/432 (35.2)	63/175 (36.0)	36/101 (35.6)	20/59 (33.9)	33/97 (34.0)

Because I am afraid of transmitting influenza to my family	147/432 (34.0)	61/175 (34.9)	33/101 (32.7)	20/59 (33.9)	33/97 (34.0)

Because I am elderly and/or have have a chronic disease	93/432 (21.5)	36/175 (20.6)	24/101 (23.8)	12/59 (20.3)	21/97 (21.6)

Why do not you undergo influenza vaccination?					

I am not afraid of influenza	775/1,711 (45.3)	329/720 (45.6)	139/314 (44.2)	128/276 (46.4)	179/401 (44.6)

I am concerned about vaccine Efficacy	511/1,711 (29.9)	211/720 (29.3)	101/314 (32.2)	80/276 (29.0)	119/401 (29.7)

I am concerned about side effects	240/1,711 (14.0)	101/720 (14.0)	43/314 (13.7)	39/276 (14.1)	57/401 (14.2)

Forgetfulness	145/1,711 (8.5)	61/720 (8.5)	25/314 (8.0)	23/276 (8.3)	36/401 (9.0)

I am against vaccinations	40/1,711 (2.3)	18/720 (2.5)	6/314 (1.9)	6/276 (2.2)	10/401 (2.5)

Have you ever received influenza vaccination before this winter season?					

Yes	643 (30.0)	264 (29.5)	141 (33.9)	96 (28.7)	142 (28.5)

Where/from whom would you like to receive influenza vaccination?					

From hospital prevention service	1,165 (54.4)	430 (48.1)°^	240 (57.8)	196 (58.5)	299 (60.0)

On the ward where I work	616 (28.8)	301 (33.6)°	110 (26.5)	89 (26.6)	116 (23.3)

At the vaccination center near my Home	138 (6.4)	55 (6.1)	29 (7.0)	21 (6.2)	33 (6.7)

From medical friends	146 (6.8)	75 (8.4)	20 (4.8)	20 (6.0)	31 (6.2)

I do not want to be vaccinated	78 (3.6)	34 (3.8)	16 (3.9)	9 (2.7)	19 (3.8)

Only one answer could be given to each question. Percentages in parentheses. HCW = healthcare worker. *p < 0.05 *vs *Emergency Department; ^*p *< 0.05 *vs *Surgery and Emergency Departments; °*p *< 0.0001 *vs *Services Department; there were no other significant differences. Only one answer could be given to each question.

The preferred source of a future vaccination would be the hospital prevention service (with a frequency ranging from 48.1% among the HCWs in the Medicine Department to 60.0% among those in the Services Department), followed by the ward on which the HCWs work (with a frequency ranging from 23.3% among the HCWs in the Services Department to 33.6% among those in the Medicine Department).

Table 2 summarizes the HCWs' opinions concerning the severity and epidemiology of influenza. The majority of HCWs (but only a minority in the Surgery Department) considered influenza a potentially serious disease. Although most HCWs in all Departments were aware of the high risk of influenza-related complications in the elderly, only some knew that schoolchildren show the highest documented incidence of the disease during the epidemic period every year (significantly fewer in the Emergency and Surgery Departments). Moreover, despite there is no prior research to show that fear of avian influenza affects vaccination rate, our data demonstrate that a limited number of respondents declared a fear of avian influenza, with the highest percentage in the Services Department. Also in this case, results were similar comparing different job categories as well comparing the different wards in each Department.

**Table 2:** Healthcare workers' opinions concerning influenza severity and epidemiology.

Questions and answers	Overall results (No. = 2,143)	Medicine (No.= 895)	Surgery (No.= 415)	Emergency (No.= 335)	Services (No.= 498)
Do you consider influenza a potentially severe disease?					

Yes	1,214 (56.6)	546 (61.0)*	203 (48.9)	198 (59.1)°	267 (53.6)

Which age group has the highest incidence of influenza-related complications?					

Children aged <2 years	236 (11.0)	86 (9.6)	51 (12.3)	39 (11.6)	60 (12.1)

Schoolchildren	97 (4.5)	46 (5.1)	19 (4.6)	10 (3.0)	22 (4.4)

The elderly	1,810 (84.5)	763 (85.3)	345 (83.1)	286 (85.4)	416 (83.5)

Which age group has the highest incidence of influenza cases?					

Children aged <2 years	285 (13.3)	99 (11.1)^	53 (12.8)	46 (13.7)	87 (17.5)

Schoolchildren	887 (41.4)	344 (38.4)°°	181 (43.6)	111 (33.1)°°	251 (50.4)

The elderly	971 (45.3)	452 (50.5)°°	181 (43.6)	178 (53.2)	160 (32.1)

Are you afraid of avian influenza?					

Yes	247 (11.5)	96 (10.7)^	50 (12.0)	27 (8.1)^	74 (14.8)

Only one answer could be given to each question. Percentages in parentheses. *p < 0.05 *vs *Surgery, Emergency and Services Departments; °*p *< 0.05 *vs *Surgery Department; ^*p *< 0.05 *vs *Services Department; °°*p *< 0.0001 *vs *Surgery and Services Departments; there were no other significant differences.

Table 3 shows the HCWs' knowledge of Italian Ministry of Health recommendations concerning influenza prevention. Considering that all of the high-risk categories listed in Table 3 are included in the Italian recommendations, if everyone got it right, 100% of the respondents should have selected each category. Only some knew all of the high-risk categories for which influenza prevention is recommended, and very few from all Departments correctly identified the categories of metabolic diseases, chronic renal dysfunction or long-term aspirin therapy. The majority knew that influenza vaccination is recommended for people aged ≥ 65 years, but their frequency was significantly lower in the Surgery Department than in the Medicine Department. Only about 70% of the HCWs in all Departments knew that they themselves were a high-risk group for which vaccination is recommended. Again, results were similar comparing different job categories as well comparing the different wards in each Department.

**Table 3:** Healthcare workers' knowledge of Italian Ministry of Health recommendations concerning influenza prevention.

Questions and answers	Overall results (No. = 2,143)	Medicine (No.= 895)	Surgery (No.= 415)	Emergency (No.= 335)	Services (No.= 498)
For which high-risk categories is influenza prevention strongly recommended?					

Asthma	1,374 (64.1)	570 (63.7)	279 (67.2)	210 (62.7)	315 (63.3)

Chronic pulmonary diseases	1,728 (80.6)	752 (84.0)	325 (78.3)*	286 (85.4)	365 (73.3)*

Hemodynamically significant cardiac diseases	1,188 (55.4)	509 (56.9)	234 (56.4)	180 (53.7)	265 (53.2)

Metabolic diseases	665 (31.0)	308 (34.4)	130 (31.3)	109 (32.5)	118 (23.7)

Chronic renal dysfunction	457 (21.3)	210 (23.5)^	92 (22.2)	60 (17.9)	95 (19.1)

Sickle cell anemia and other hemoglobinopathies	1,164 (54.3)	485 (54.2)	232 (55.9)	188 (56.1)	259 (52.0)

Immunosuppressive disorder or therapy	1,140 (53.2)	476 (53.2)	219 (52.8)	185 (55.2)	260 (52.2)

Long-term aspirin therapy	141 (6.6)	48 (5.4)	37 (8.9)	20 (6.0)	36 (7.2)

People aged ≥ 65 years	1,681 (78.4)	732 (81.8)°	302 (72.8)	261 (77.9)	386 (77.5)

HCWs	1,503 (70.1)	641 (71.6)	279 (67.2)	238 (71.0)	345 (69.3)

Multiple answers were allowed. Percentages in parentheses. HCWs = healthcare workers. *p < 0.05 *vs *Medicine and Emergency Departments; ^*p *< 0.05 *vs *Emergency Department; °*p *< 0.05 *vs *Surgery Department; there were no other significant differences.

Table 4 shows the HCWs' knowledge of influenza vaccines and antiviral drugs. The majority were unable to indicate the types of influenza virus included in the vaccine, with significantly less being known by the HCWs in the Emergency Department. Furthermore, most did not know which drugs were registered for influenza prevention and treatment, with significantly less knowledge being shown by the HCWs in the Emergency and Services Departments. Answers were similar comparing different job categories as well comparing the different wards in each Department.

**Table 4:** Healthcare workers' knowledge of influenza vaccines and antiviral drugs.

Questions and answers	Overall results (No. = 2,143)	Medicine (No.= 895)	Surgery (No.= 415)	Emergency (No.= 335)	Services (No.= 498)
How many viruses are included in influenza vaccine?					

I do not know	1,678 (78.3)	679 (75.9)	322 (77.6)	282 (84.2)*	395 (79.3)

A H1N1	322 (15.0)	149 (16.6)	72 (17.3)	35 (10.4)°	66 (13.2)

A H3N2	266 (12.4)	119 (13.3)	53 (12.8)	32 (9.6)	62 (12.4)

A H5N1	127 (5.9)	53 (5.9)	37 (8.9)	13 (3.9)°	24 (4.8)

B	131 (6.1)	62 (6.9)	24 (5.8)	11 (3.3)^	34 (6.8)

C	54 (2.5)	23 (2.6)	11 (2.7)	5 (1.5)	15 (3.0)

What drugs are registered for influenza prevention?					

I do not know	1,788 (83.4)	706 (78.9)"	351 (84.6)	294 (87.8)	437 (87.7)

Amantadine	184 (8.6)	113 (12.6)**	34 (8.2)	18 (5.4)	19 (3.8)

Rimantadine	84 (3.9)	46 (5.1)°°	13 (3.1)	13 (3.9)	12 (2.4)

Zanamivir	167 (7.8)	81 (9.1)	26 (6.3)	23 (6.9)	37 (7.4)

Oseltamivir	62 (2.9)	33 (3.7)	7 (1.7)	6 (1.8)	16 (3.2)

What drugs are registered for influenza therapy?					

I do not know	1,805 (84.2)	725 (81.0)**	346 (83.4)	294 (87.8)	440 (88.3)

Amantadine	166 (7.7)	88 (9.8)°°	32 (7.7)	21 (6.3)	25 (5.0)

Rimantadine	79 (3.7)	40 (4.5)	14 (3.4)	10 (3.0)	15 (3.0)

Zanamivir	175 (8.2)	86 (9.6)	33 (8.0)	22 (6.6)	34 (6.8)

Oseltamivir	71 (3.3)	35 (3.9)	11 (2.7)	8 (2.4)	17 (3.4)

Multiple responses possible other than "I do not know". Percentages in parentheses. *p < 0.05 *vs *Medicine, Surgery and Services Departments; °*p *< 0.05 *vs *Medicine and Surgery Departments; ^*p *< 0.05 *vs *Medicine and Services Departments; "*p *< 0.05 *vs *Surgery, Emergency and Services Departments; **p < 0.05 *vs *Emergency and Services Departments; °°*p *< 0.05 *vs *Services Department; there were no other significant differences.

Table 5 summarises the multivariate analysis of the associations between the use of influenza vaccination before the 2005–2006 influenza season and HCWs' characteristics, knowledge and attitudes. The factors positively associated with being vaccinated were an age of ≥ 45 years, considering influenza a potentially severe disease, and being aware of all of the high-risk categories for which influenza vaccination is strongly recommended; the factors negatively associated with being vaccinated were being a female, working in the Medicine Department, and being nurses or paramedics.

**Table 5:** Multivariate analysis of the associations between influenza vaccination received before the 2005–2006 influenza season and the healthcare workers' characteristics, knowledge and attitudes.

Factors	Crude OR (95% CI)	OR (95% CI)*	*P *value
Gender			

Males	1°	1°	

Females	0.60 (0.50–0.73)	0.76 (0.61–0.95)	0.016

Age			

<25 yrs	1°	1°	

25–34 yrs	1.07 (0.66–1.74)	0.97 (0.59–1.60)	0.90

35–44 yrs	1.27 (0.79–2.06)	1.46 (0.87–2.44)	0.15

45–54 yrs	1.83 (1.14–2.95)	1.92 (1.14–3.23)	0.015

≥ 55 yrs	2.99 (1.79–4.97)	2.80 (1.60–4.90)	<0.001

Department			

Surgery	1°	1°	

Medicine	0.76 (0.58–1.01)	0.68 (0.50–0.92)	0.013

Emergency	0.67 (0.47–1.06)	0.68 (0.46–1.04)	0.085

Services	0.76 (0.55–1.04)	0.72 (0.51–1.01)	0.06

Type of employment			

Physicians	1°	1°	

Nurses	0.52 (0.43–0.64)	0.54 (0.42–0.68)	<0.001

Paramedics	0.51 (0.37–0.70)	0.48 (0.33–0.69)	<0.001

Influenza considered a potentially severe disease			

No	1°	1°	

Yes	1.95 (1.61–2.37)	1.64 (1.32–2.03)	<0.001

Awareness of all of the high-risk categories for which influenza prevention is strongly recommended			

No	1°	1°	

Yes	2.42 (1.91–3.06)	2.24 (1.74–2.88)	<0.001

*The odds ratios (OR) and 95% confidence intervals (CI) were estimated using multivariate logistic regression. °Reference category. Only the factors with a *p *value of < 0.05 are shown.

## Discussion

The data described in this paper came from a survey carried out in one of Italy's largest multidisciplinary University Hospitals and included a large number of HCWs from all specialties. Results show that influenza vaccination coverage was less than 25% in all of the Departments and, ironically, its prevalence was lowest in the Emergency Department, the area to which patients at highest risk of influenza complications are admitted and in which HCWs have the greatest risk of exposure to influenza from patients. Moreover, compliance was poor among all of the HCW categories, but worse among the nurses and paramedics. This non-compliance with official recommendations concerning the use of influenza vaccine [7-9] suggests that, despite some minor but significant differences between the specialties, this is a basic problem involving all types of HCWs.

This is the first comprehensive study on influenza vaccination among HCWs conducted in Italy and to the best of our knowledge one of the first studies that compares the personal use of influenza vaccination in different Departments within a hospital and in different types of HCWs. Moreover, our study sample was larger than that evaluated in previous reports [17-24] and, probably because of the active involvement of our hospital's Health Management Unit, we obtained an excellent participation rate.

Our data support the findings of other recent studies [17-23], although our coverage rate is one of the lowest. This can be explained bearing in mind that influenza vaccine has always been offered free of charge in our hospital, but without any information/educational campaigns and without any incentive. A number of recent studies have clearly demonstrated that the highest level of coverage is reached when vaccine is given free of charge after extensive educational programs and in association with incentives [25-29].

Our data also confirm that a lack of knowledge of influenza and its related complications affects the vaccination rate of HCWs [24,30]. This is demonstrated by the lack of knowledge regarding the severity of influenza in relation to age and subjects at high risk when infected by influenza viruses, the current recommendations on influenza prevention (what they are and what they mean) as well as by the results of the multivariate analysis. These findings suggest that there is a pressing need for educational programs aimed at removing the barriers that limit compliance to official recommendations. A number of methods have been suggested to improve HCW influenza vaccination rates, but they can only be fully successful if hospital management (owners, administrators, and medical directors) is convinced that vaccinating their employees against influenza is important for both medical and economic reasons. It has been clearly demonstrated that HCWs can transmit influenza to their patients, thus causing a significant increase in morbidity and mortality [11-13], and model-based economic analyses have shown that influenza vaccination is largely cost-effective because it reduces employee absenteeism and related problems during influenza outbreaks [14-16].

Whatever educational method is adopted to increase the knowledge of influenza, it needs to be applied to all HCWs, although particular attention has to be paid to some specific problems. Active and organized hospital vaccination campaigns that consider factors such as the characteristics of the wards, main medical fields, and the occupational category of HCWs have been associated with increased vaccination coverage [27-30]. Our results confirm that HCWs would like to have convenient access to influenza vaccine at work as part of their employee health programs. In this regard, vaccination programs that combine publicity and education for HCWs, a plan for identifying the people recommended for vaccination, the use of reminder/recall systems, the assessment of practice-level vaccination rates with feedback to staff, and efforts to remove the administrative and financial barriers that prevent people from undergoing vaccination are urgently needed everywhere, regardless of HCW specializations and their hospital roles.

However, some of our findings suggest that such educational programs should concentrate particularly on some HCWs. The multivariate analysis showed that an age of ≥ 45 years, considering influenza as a potentially severe disease and being aware of influenza high-risk categories were positively associated with being vaccinated, whereas working in the Medicine Department and being nurses or paramedics were negatively associated with being vaccinated. This means that specifically tailored interventions should be planned for young HCWs, as well as nurses and paramedics. Furthermore, respondents in our study said that the preferred source of a future vaccination would be the hospital prevention service, followed by the ward on which the HCWs work. These findings should also be considered as a focus for future promotional activities.

This study is limited by the fact that the survey was conducted during a single influenza season and in a single health care facility. However, although it is necessary to conduct additional surveys during subsequent influenza seasons (and in other hospitals) before the results can be generalised, it is clear that extensive and sustained efforts to overcome the lack of knowledge about influenza and its prevention are required to ensure compliance with the current recommendations concerning the use of influenza vaccine among Italian HCWs. Moreover, the questions on the HCWs' knowledge of influenza vaccines and antiviral drugs could be considered a bit difficult. However, these answers only support our conclusions, did not influence our multivariate analysis and confirm the need of comprehensive educational program on influenza and its prevention among HCWs.

## Conclusion

Our data show that influenza vaccination coverage among Italian HCWs at a large multi-disciplinary hospital is very low. Although educational methods to eliminate the barriers that reduce compliance with official recommendations should be planned for all HCWs, the differences between Departments and types of HCWs appear significant. This identification of the HCWs who are more likely to be non-compliant, such as nurses and paramedics as well as those of the Department of Emergency, makes it possible to prepare specific and more detailed educational programs in order to obtain the best results. Extensive efforts to provide information concerning the risks and benefits of influenza immunization among HCWs, and enhance the accessibility of the vaccine, seem to be warranted in order to reduce morbidity and mortality in high-risk patients, contain nosocomial outbreaks, and ensure the greatest socioeconomic impact.

## Abbreviations

HCWs: Health care workers; Ors: Odds ratios; CIs: Confidence intervals.

## Competing interests

The authors declare that they have no competing interests.

## Authors' contributions

SE supervised the study, developed the study design, and drafted the manuscript. SB participated in acquisition of data and assisted with the interpretation of the data. CP created the database and conducted the statistical analysis. ET, CS, MS participated in acquisition of data. PM assisted with the study design and participated in the interpretation of the data. FdL assisted with the study design. NP participated in the study design, and drafted the manuscript.

## Pre-publication history

The pre-publication history for this paper can be accessed here:


